# Rising Global Opportunities Among Orthopaedic Surgery Residency Programs

**DOI:** 10.5435/JAAOSGlobal-D-20-00102

**Published:** 2020-12-14

**Authors:** Jacob Pfeifer, Noah Svec, Chandrakanth Are, Kari L. Nelson

**Affiliations:** From the College of Medicine, University of Nebraska Medical Center, Omaha, NE (Mr. Pfeifer and Mr. Svec), and the Department of Surgery, University of Nebraska Medical Center, and Office of Graduate Medical Education, Omaha, NE (Dr. Are and Dr. Nelson).

## Abstract

**Objective:**

We surveyed Orthopaedic Surgery Residency (OSR) programs to determine international opportunities by the academic institutional region within the United States, location of the international experience, duration, residency program year (PGY), funding source, and resident participation to date.

**Design:**

We emailed a survey to all OSR programs in the United States to inquire about global opportunities in their residency programs. Further contact was made through an additional e-mail and up to three telephone calls. Data were analyzed using descriptive and chi-square statistics. This study was institutional review board exempt.

**Setting:**

This research study was conducted at the University of Nebraska Medical Center, a tertiary care facility in conjunction with the University of Nebraska Medical Center College of Medicine.

**Participants:**

The participants of this research study included program directors and coordinators of all OSR programs (185) across the United States.

**Results:**

A total of 102 OSR programs completed the survey (55% response rate). Notably, 50% of the responding programs offered a global health opportunity to their residents. Of the institutions that responded, those in the Midwest or South were more likely to offer the opportunity than institutions found in other US regions, although regional differences were not significant. Global experiences were most commonly: in Central or South America (41%); 1 to 2 weeks in duration (54%); and during PGY4 or PGY5 (71%). Furthermore, half of the programs provided full funding for the residents to participate in the global experience. In 33% of the programs, 10 or more residents had participated to date.

**Conclusions:**

Interest in global health among medical students is increasing. OSR programs have followed this trend, increasing their global health opportunities by 92% since 2015. Communicating the availability of and support for international opportunities to future residents may help interested students make informed decisions when applying to residency programs.

Medical students are becoming increasingly interested in global opportunities,^1^ but information on these opportunities during residency can be difficult to find and would be especially valuable during the residency application process. Global health and global opportunities are gaining greater attention from today's surgery residents than previous generations.^1^ Many surgery programs and the Accreditation Council for Graduate Medical Education (ACGME) have recognized students' and trainees' increasing interest in global opportunities. Therefore, in 2011, the ACGME, together with the Residency Review Committee for Surgery and the American Board of Surgeons, developed parameters that allow some global rotations to count toward completion of residency.^2^

Although many surgical programs have begun offering global health experiences, there are well-documented obstacles to providing these educational opportunities, such as lack of funding, time, or engagement by faculty, among others.^3,4,5^ Furthermore, there is limited research related to the effect of global surgery programs for both the participants in the programs and the recipients of the surgical services.^6^ One study associated this lack of empirical research with a lack of engagement by academic surgeons, concluding that there has been insufficient involvement of academic surgeons to ensure the progression of research related to global surgery experiences.^6^ This lack of involvement by academic surgeons may be due to the fact that, frequently, the physicians are not compensated by their respective institutions for time or expenses to go abroad. Institutions may not be incentivized to compensate their faculty's involvement in global opportunities because going abroad may disrupt many areas of the surgeons' typical responsibilities, including generating revenue for the institution, producing curricular material, publishing, and mentoring of trainees who do not go abroad.^7^ In addition, trainees who undertake global rotations may lose out on clinical and educational experiences at their own hospitals as well as incur more financial burden.^7^

Despite the challenges of implementing global surgery programs, the need for surgeons in low- to middle-income countries (LMICs) continues to be notable. In 2015, the Lancet Commissions published a manuscript outlining a large-scale plan for global surgery for the year 2030.^8^ The outline included five key messages to improve access to surgical and anesthesia care worldwide: (1) Five billion people do not have access to timely and safe surgical care; (2) 143 million additional surgical procedures are needed each year to save lives; (3) 33 million people receive surgical care but now face debilitating financial burden; (4) investing in surgical services in the lower income countries is economically feasible; and (5) surgery is a vital part of health care that needs to be involved in all health systems.^8^ Clearly, there is a compelling need for surgical specialties to serve in LMIC.

Although the need for surgical care internationally is clear, this parallels potentially valuable opportunities for residents to add unique breadth to their training. During global rotations, residents can participate in cases related to diseases that may be less prevalent in the United States or the area where they currently practice. Previous studies suggest these unique cases and disease states pose ample opportunities in teaching and research.^6^ In a study by Henry et al,^9^ the benefits of international rotations for US Surgery residents were evaluated, and they found that the benefits included: Learning to work with limited resources, exposure to diverse surgical pathology, experiencing a new culture, and creating rapport with local medical leaders in the community. Subsequently, Dr. Frank Lewis, Executive Director, American Board of Surgery, provided a commentary on the study by Henry et al, where he points out that the respondents provided a large list of potential benefits but failed to come to agreement regarding the main benefits of an international surgical experience. He emphasizes that only a few items in their study receive greater than 9% agreement, leading him to believe that the perceived benefits are diverse and lack consensus. In his commentary, he argues that the notable benefits to residents can include but are not limited to the development of a one-to-one relationship with the patient, the opportunity to learn from a large diverse caseload in short time, and the ability to function in a setting where one does not have stringent time constraints.^10^

Despite a lack of consensus on the benefits of global surgery experiences, most studies agree that residency programs hold unique potential relative to the global surgery needs. Although there are numerous obstacles to creating and sustaining global health experiences through residency programs, many programs have taken on this challenge and are currently providing these experiences. For example, the availability of global rotations in ophthalmology,^11^ general surgery,^12^ and orthopaedic surgery^13^ has been previously documented at 54%, 34%, and 26%, respectively. At 26%, Orthopaedic Surgery Residency (OSR) programs provide a relatively low rate of international health experiences to their residents. However, these findings are from 2015 or earlier and because of a rapidly changing landscape and demand, the data need to be updated to provide current information.

Furthermore, since 2015, the total number of OSR programs in the United States has increased by more than 30 programs.^14^ Because of the growing number of OSR programs and the lack of research in the area of global health electives within residency programs, the aim of this research was to help bridge the gap of information and provide a more comprehensive and current view of international opportunities within OSR programs. Specifically, we examined OSR programs to determine international opportunities by the institutional region, location of the international experience, duration, resident participation to date, and funding source.

## Methods

For this study, we used the American Medical Associations' Fellowship and Residency Electronic Interactive Database (FREIDA)^14^ to compile a list of the 185 OSR programs in the United States. FREIDA is a freely accessible, online database of all graduate medical education programs that are accredited by the ACGME. Our compiled list included the name of the program, as well as the program director and the program coordinator with his/her contact information. An initial e-mail was sent out to all 185 program coordinators, which explained our investigation into global health opportunities in OSR programs and included a survey link (Supplemental Material 1, http://links.lww.com/JG9/A106). The institutional review board deemed the study exempt because the data collected did not involve human subjects.

To develop the survey, the researchers conducted a literature review and found three studies in particular (conducted between 2015 and 2016), in general surgery and orthopaedic surgery, with objectives that aligned to those of our study.^2,12,13^ Using this literature, input from a former surgery program director and an expert education researcher, we refined the questions and reduced the content down to seven fixed-response questions and an open-ended response option for additional comments. Our final survey was similar to the survey from Sobral et al, we intentionally kept the survey brief to help ensure a robust, national response. We specifically defined a global health or international experience as a time where a US Orthopaedic Surgery resident traveled outside of the continental United States to provide medical care. This included mission trips, formal elective rotations, experiences planned during vacation time, and global health tracks.

After this initial e-mail, we gave the program coordinators 1 week to fill out the survey. Once the week had passed, an additional e-mail was sent out to the programs that had not responded. If the program did not respond to the second e-mail, we began personally calling the program coordinators or program directors. When contact was made with the program, they were provided information about the study and were asked if they would like to participate. Those who were willing to participate were asked the seven questions from the survey. If the programs were unwilling to participate, they were marked as “no response.” Programs that were unable to be reached after three separate phone calls were also marked as “no response.” Data were analyzed using descriptive statistics and chi-square analysis.

## Results

Of the 185 OSR programs listed on the FREIDA^14^ database, 102 programs responded to the survey through either e-mail or telephone call, resulting in a response rate of 55%. Of the 102 programs that responded, 51 (50%) offered a global health opportunity during residency training (see Table 1 for a list of programs), and 51 did not offer any experience. In total, 83 programs (45%) did not respond by e-mail or were unable to be reached after three telephone calls and were deemed a “no response.”

**Table 1:** Orthopaedic Surgery Residency Program Survey Respondents That Offer a Global Health Experience

Northeast	Midwest	South	West
Monmouth Medical Center	Summa Health System/NEOMED	University of Mississippi Medical Center	University of California (San Francisco)
Penn State Health Milton S Hershey Medical Center	Kettering Health Network	Duke University Hospital	University of Arizona college of Medicine-Tucson
Zucker School of Medicine at Hofstra/Northwell	Midwest School^a^	Wake Forest University School of Medicine	University of New Mexico School of Medicine
New York-Presbyterian Hospital	University of Missouri-Kansas City School of Medicine	John Peter Smith Hospital (Tarrant County Hospital District)	University of Hawaii
UPMC Medical Education (Hamot)	University of Missouri-Columbia	Johns Hopkins University	University of California Davis Health
York Hospital	University of Minnesota	University of Texas Health Science Center San Antonio	Loma Linda University Health Education Consortium
St Joseph's University Medical Center	Metro Health University of Michigan Health (Metro Health)	West Virginia University	Community Memorial Health System
New York University School of Medicine/Hospital for Joint Diseases	Beaumont Health (Royal Oak and Taylor)	Medical College of Georgia	
University of Rochester	Mayo Clinic College of Medicine and Science (Rochester)	Jackson Memorial Hospital/Jackson Health System	
Albert Einstein Healthcare Network	Cleveland Clinic Foundation/South Pointe Hospital	University of Virginia Medical Center	
University of Connecticut	Beaumont Health (Farmington Hills and Dearborn)	University of Florida College of Medicine Jacksonville	
Brown University	Loyola University Medical Center	University of Tennessee College of Medicine at Chattanooga	
	Henry Ford Hospital/Wayne State University	University of Tennessee	
	Medical College of Wisconsin Affiliated Hospitals	University of Texas Health Science Center Houston	
	Western Michigan University Homer Stryker MD School of Medicine	University of Kentucky College of Medicine	
	Henry Ford Macomb Hospital	University of Texas at Austin Dell Medical School	

a School requested that they remain unnamed.

The programs that responded were grouped based on geography, using the US Census Bureau map (Supplemental Material 2, http://links.lww.com/JG9/A106), which divides the United States into Northeast, South, Midwest, and West regions.^15^ Of the programs that responded, the Midwest region had the greatest percentage of programs with global rotations (54%), whereas the Northeast region had the lowest percentage (43%) (Table 2). However, using chi-square analysis, global offerings by the OSR program region were not significantly different (χ^2^ = 0.66, df = 3, N = 102, *P* > 0.05).

**Table 2:** Global Opportunities by the Region of the United States

Region	Responding Programs (N)	Global Opportunity Per Region (%)	Global Opportunity % Per Total Affirmative Respondents
Midwest	30	16 (55)	32
Northeast	28	12 (43)	24
South	32	16 (50)	32
West	13	7 (54)	14
Total	102	51 (50)	100

**Figure 1:**
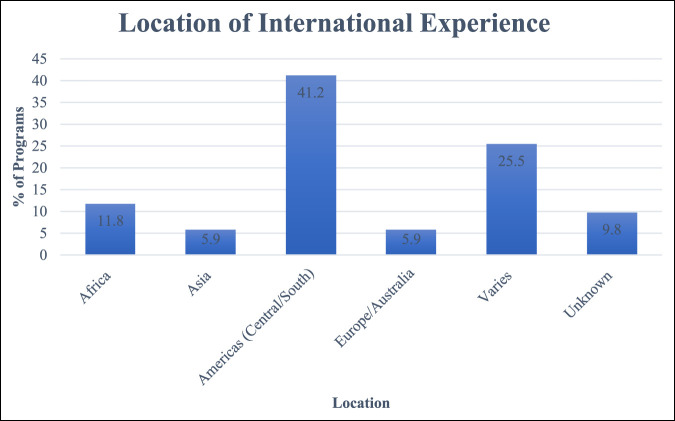
Bar chart showing the most common international experiences by location. Experiences in the Americas (Central and South America) were the most common in programs across Orthopaedic Surgery Residencies.

For the programs that offered global opportunities, we examined the locations of these experiences. We found that 21 of the 51 programs (about 41%) had global health experiences located in Central and South America, making the Americas the most common site. About 25% (13/51) of programs had locations that varied, leaving it up to the resident to go wherever they chose (Figure 1).

**Figure 2:**
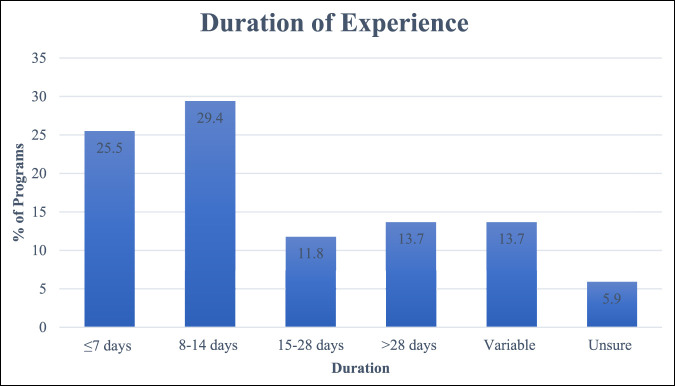
Bar chart showing the average length of global experiences for Orthopaedic Surgery Residency survey respondents. Most programs were 1 to 2 weeks in duration.

While on a global health experience, the most common duration of the trip was between 1 and 2 weeks. Of the 51 programs that responded, 15 (29.4%) stated that the experience lasted 1 to 2 weeks, whereas 13 (25.5%) had a global experience lasting 1 week or less. The most common years for participation were residency program year 4 and 5, with 71% (36/51) of programs stating these were the preferred years (Figure 2).

**Figure 3:**
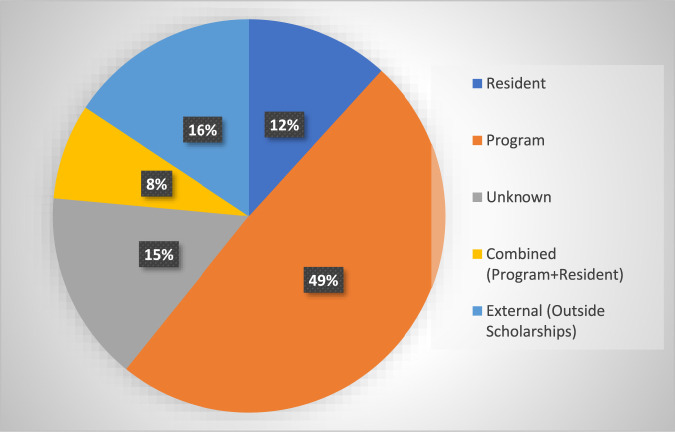
Pie chart showing funding sources for international experiences in Orthopaedic Surgery Residency programs. About one-half of the programs pay in full for the experience.

Furthermore, one-third (17/51) of the programs have sent greater than 10 residents on a global health experience, whereas only 6% of respondents with global opportunities (3/51) have sent no residents (Table 3). Interestingly, 49% of the programs fund the global health experience through the program itself, and 12% (6/51) have the residents pay out of pocket (Figure 3).

**Table 3:** Orthopaedic Surgery Resident Participation to Date Per Respondent Program

No. of Residents	Programs (%)
Unknown	5 (9.80)
0	3 (5.88)
1	9 (17.65)
2-4	5 (9.80)
5-10	17 (33.33)
≥10	12 (23.53)

## Discussion

Medical students around the country have an increased desire to participate in global health rotations. In a recent study, 66% of medical student respondents in the United States reported an interest in global health opportunities.^1^ With this growing interest from future physicians, we hypothesized that residency programs would also have increasing opportunities in global health experiences. In OSR programs specifically, a 2015 study indicated that only 26% of responding programs offered a global health experience/international elective in OSR training.^13^ Since that time, the number of OSR programs in the United States has grown from 154 to 185.^14^ Along with this rapid growth in programs, there has also been an increase of global health opportunities during residency, noting that 50% of program respondents to our survey offered such an experience. Taken together, since 2015, OSR programs have grown by about 17%, whereas global health opportunities within this specialty have grown by 92%. This incredible growth in just 4 years is very encouraging and provides increased opportunities to the future orthopaedic residents who have an interest in global health.

In our breakdown of regions, it was found that the Midwest region had the greatest percentage of OSR programs offering a global health opportunity to their residents; however, this difference was not notable. Therefore, students who are interested in working abroad during residency need not focus on institutions in one region over another, rather they should investigate the individual programs.

In terms of destination for the rotation, although our study found Central and South America were the most common sites for current orthopaedic global health opportunities, there are opportunities all over the world. Previous studies have shown that, for international elective opportunities in many specialties, Africa offers the greatest number of options, with the Americas having the second greatest number of opportunities. The next most popular sites are the Western Pacific and Southeast Asia. Europe and the Mediterranean typically offer the smallest number of global health experiences.^16^ Notably, Kerry et al^16^ found there was a statistically significant correlation between the burden of disease in a country and the number of global health opportunities offered. Although global programs for residents can be anywhere, they tend to be in areas with notable mortality and morbidity.

Most programs indicated that their global rotations are 1 to 2 weeks on average. Although this may be a relatively short timeframe, previous studies indicate that short-term electives can still be beneficial for residents. These rotations often have a surgical volume that exceeds what residents would have had at their institution during the same timeframe. In addition, even short international electives can provide experiences that meet the requirements of the resident's training.^17^ Specifically, in the United States, residents must attain the six core competencies of the ACGME. These six competencies are patient care, medical knowledge, practice-based learning and improvement, interpersonal and communication skills, professionalism, and systems-based practice.^18^

The likelihood of meeting these competencies is reinforced by the fact that residents must apply to participate in an international rotation. The application process can vary from one institution to another, involving factors internal and external to the program. The internal factors are primarily related to the ability of a program to establish the rotation. From our own experience and knowledge, most programs permit residents to apply based on their individual interest, and these residents are typically allowed to complete the international rotation. The specific criteria used by any individual program are beyond the purview of this study. The external criteria that need to be satisfied are those set forth in the Common Program Requirements from the ACGME.^19^

Beyond meeting ACGME competencies, previous studies have detailed other benefits of participating in global rotations. In a study conducted at the Mayo Clinic, residents stated their global experiences exposed them to unique pathology not usually seen in the United States. In addition, residents had to learn how to manage limited resources and, in some cases, explore alternate treatment options. Unable to always rely on diagnostic testing, the residents were also able to hone their physical examination and history-taking skills. Finally, residents stated they were exposed to different cultures and were challenged to develop critical thinking skills.^20^

Another reason to be encouraged by the findings of the current study is that 33% of respondent programs had sent more than 10 residents on global health experiences. Programs that have sent multiple residents on international rotations can serve as models for programs that are looking to expand their international options for their residents. In a previous study by Matar et al,^21^ nearly half of surgery residents stated that the availability of international health electives would have positively influenced their residency program, so developing these global rotations may promote interest in residency programs.

A factor that makes global health experiences more manageable and attractive to residents and medical students considering residency programs is the fact that in our study, about half of the OSR programs offering such an experience pay in full for the resident to go abroad, which has previously been noted as a major barrier to international programs. Furthermore, an additional 8% of programs cover a portion of the costs for their residents. Because residents matriculating out of the medical school have an average of nearly $200,000 in student loans,^22^ it is encouraging that most programs in our study assume at least some of the financial burden of these global experiences.

Notably, half of the OSR program respondents to our survey still do not offer a global health opportunity. However, the rapid growth that was seen in global health opportunities in this specialty is very encouraging for those who wish to participate during residency. Global health opportunities should continue to be monitored and studied to ensure maximum benefit for both the patients receiving the care and the resident.

This study has several limitations. The 55% response rate, while robust for medical education research, also suggests that the results are not necessarily generalizable. The results are limited to the program directors/coordinators who responded to the survey, and the true number of OSR programs offering global health opportunities cannot be known without full participation. Programs that do not offer a global health opportunity may be less likely to respond to the survey, potentially leading to a response bias. In addition, by asking program directors and coordinators to answer survey questions to the best of their abilities, there is room for human error and subjectivity.

The number of OSR programs in the United States is growing and global health opportunities in OSR mirror this trend. As the need for and interest in providing health care abroad increase, programs should continue to find ways to overcome the factors that limit resident participation in other countries, especially LMIC. Current trajectories of participation and funding for global opportunities in OSR are encouraging. Residency programs should make detailed information about such opportunities in their programs readily available so that future orthopaedic residents who are interested can make informed decisions.

## Supplementary Material

SUPPLEMENTARY MATERIAL
