# Supplementary material for: Rising Global Opportunities Among Orthopaedic Surgery Residency Programs
Source: J Am Acad Orthop Surg Glob Res Rev. 2020 Dec 14;4(12):e20.00102. doi: 10.5435/JAAOSGlobal-D-20-00102 (PMC7738025; doi:10.5435/JAAOSGlobal-D-20-00102)

Supplemental Material 1. Survey questions with the definition of an International Experience and the survey link.

International Experience: a time when a US Orthopaedic Surgery Resident traveled outside of the continental US to provide medical care. This includes mission trips, formal elective rotations, experiences planned during vacation time, and global health tracks.

1. What is the name of the program you are representing?

1. Do you offer a global health elective in your residency program?

1. What is the location of the global health elective? (please list all)

1. How long does the elective last? (if there are different options, please list all)

1. What year of residents participate in the international experience? (list all)

1. What is the funding source of the international experience?

1. How many residents have participated in the international experience to date?

1. Additions Comments

Supplemental Material 2. United State Census Bureau map dividing the country into five different regions based on location. <https://www.2.census.gov/geo/pdfs/maps-data/maps/references/us_regdiv.pdf>


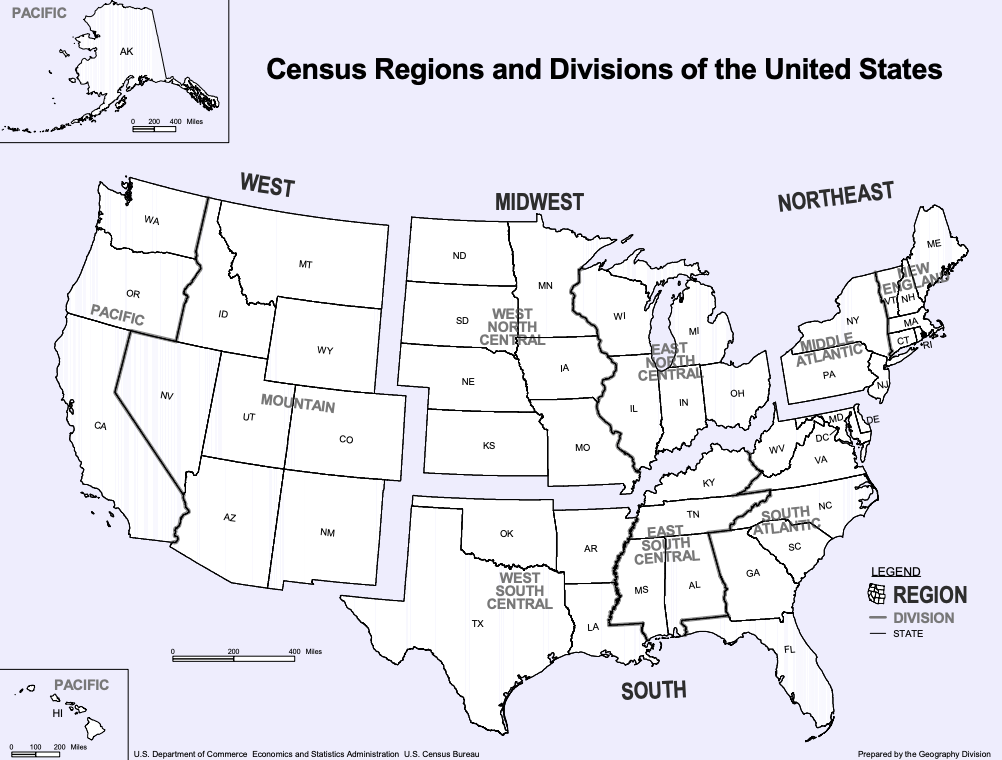

Supplement: SUPPLEMENTARY MATERIAL [file jagrr-4-e20.00102-s001.docx]
